# What predicts adolescents’ mathematics anxiety in the Middle East and North Africa? An interpretable machine learning analysis of six Arab education systems in PISA 2022

**DOI:** 10.3389/fpsyg.2026.1929157

**Published:** 2026-09-09

**Authors:** Omar Allohibi

**Affiliations:** 1 Department of Educational Policies and Economics, College of Education, Taibah University, Madinah, Saudi Arabia; 2 Energy, Industry, and Advanced Technologies Research Center, Taibah University, Madinah, Saudi Arabia

**Keywords:** adolescents, control-value theory, machine learning, mathematics anxiety, MENA, PISA 2022

## Abstract

**Introduction:**

Mathematics anxiety is among the most consequential affective barriers to mathematics learning, yet large-scale evidence from the Middle East and North Africa (MENA) remains scarce. Existing research on the region rarely moves beyond linear models, and no study has applied interpretable machine learning (ML) to identify which psychological, instructional, and contextual factors jointly predict mathematics anxiety among Arab adolescents.

**Methods:**

Drawing on the Programme for International Student Assessment (PISA) 2022, we analysed 47,652 fifteen-year-old students from the six participating Arab education systems: the United Arab Emirates, Jordan, Morocco, Palestine, Qatar, and Saudi Arabia. Six algorithms (linear regression, elastic net, random forest, AdaBoost, XGBoost, and LightGBM) were tuned with randomized search under 5-fold cross-validation to predict the PISA mathematics anxiety index (ANXMAT) from 50 predictors spanning competence beliefs, engagement, instructional climate, social relationships, achievement, and demographics. SHapley Additive exPlanations (SHAP) values, computed exactly for the selected gradient-boosting model, quantified the magnitude, direction, and heterogeneity of each predictor’s contribution, complemented by gender- and country-stratified decompositions and five robustness analyses.

**Results:**

XGBoost achieved the best cross-validated performance (CV *R*^2^ = 0.275; test *R*^2^ = 0.266, RMSE = 0.978), outperforming linear benchmarks by approximately 10% in explained variance. Mathematics self-efficacy was the dominant predictor, followed by effort and persistence in mathematics, self-efficacy for mathematical reasoning, and mathematics achievement. Growth mindset ranked fifth despite a near-zero bivariate correlation, indicating a conditional predictive association that marginal analyses conceal. Girls reported systematically higher predicted anxiety, and while the protective core (self-efficacy, persistence) operated near-identically across genders, the anxiety penalty associated with bullying victimization was almost twice as steep for girls. The predictor hierarchy replicated across all six systems (mean pairwise rank correlation = 0.88).

**Discussion:**

The findings are consistent with control-value theory (CVT): perceived competence, not socioeconomic status or resources, is the primary correlate of adolescents’ mathematics anxiety in MENA. These cross-sectional findings are predictive, not causal; within the fitted model, the gender-asymmetric social stressors and the region-wide consistency of the predictive architecture identify efficacy-building instruction, anxiety-sensitive homework design, and anti-bullying policy as priority candidates for intervention research across Arab education systems.

## 1 Introduction

Few affective experiences shape adolescents’ relationship with mathematics as profoundly as mathematics anxiety: the tension, apprehension, and fear that many students feel when they anticipate, perform, or even merely think about mathematical tasks (Dowker et al., 2016; Richardson and Suinn, 1972). Far from a transient discomfort, mathematics anxiety consumes working-memory resources during problem solving (Ashcraft and Kirk, 2001), triggers avoidance of mathematically demanding courses and careers (Meece et al., 1990), and shows robust negative associations with achievement across hundreds of studies and virtually every education system examined (Barroso et al., 2021; Foley et al., 2017; Namkung et al., 2019). Because mathematical competence increasingly gatekeeps access to science, technology, engineering, and mathematics (STEM) trajectories in digitalising economies, understanding what predicts mathematics anxiety is not merely a psychological question but an economic and social one.

This question is particularly urgent, and particularly under-researched, in the Middle East and North Africa (MENA). Six Arab education systems participated in the PISA 2022: Jordan, Morocco, Palestine, Qatar, Saudi Arabia, and the United Arab Emirates. These systems share an uncomfortable profile: mathematics achievement substantially below the OECD average, despite exceptional levels of educational investment in the Gulf states (OECD, 2023), combined with levels of mathematics anxiety at or above the OECD average. In the pooled sample analysed here, all six systems scored above the OECD mean on the PISA mathematics anxiety index. The region also presents a theoretically provocative gender configuration: whereas boys outperform girls in mathematics across most OECD systems, gender achievement gaps in the Arab systems are small, absent, or reversed in girls’ favor, and girls outperform boys in reading by wide margins (El-Kogali and Krafft, 2019; OECD, 2023). Whether the well-documented female excess in mathematics anxiety (Devine et al., 2012; Else-Quest et al., 2010) is replicated under these conditions, and whether anxiety is driven by the same factors among girls and boys, are open questions with direct policy implications for a region undergoing rapid, education-centred economic transformation.

Methodologically, research on the antecedents of mathematics anxiety has relied overwhelmingly on linear regression and structural equation models applied to small or single-country samples (Luttenberger et al., 2018). These approaches presuppose additive, linear relationships and require the analyst to pre-select a small set of predictors, leaving two blind spots. First, they cannot easily reveal which of the many correlated candidate factors—competence beliefs, motivation, instructional climate, peer relationships, family resources, and achievement itself—carry unique predictive weight when all are considered jointly. Second, they conceal non-linear and conditional effects, such as protective factors that matter only for vulnerable subgroups. Machine learning (ML) methods, particularly gradient-boosted tree ensembles, address both limitations by learning flexible functional forms from data and by handling large, collinear predictor sets without *a priori* exclusion (Hastie et al., 2009; Yarkoni and Westfall, 2017). Combined with SHAP values, which decompose each individual prediction into exact, additive feature contributions (Lundberg and Lee, 2017; Lundberg et al., 2020), this yields an analytical strategy that is simultaneously predictive and interpretable.

A rapidly growing literature has applied this ML-plus-SHAP paradigm to international large-scale assessment data, but almost exclusively to predict cognitive outcomes: mathematics achievement in East Asia (Djekourmane et al., 2026; Wu, 2024), STEM competencies across the full PISA 2022 sample, or academic resilience among disadvantaged students (Cheung et al., 2024). Affective outcomes have remained on the margins. The closest precedent used penalized linear regression, not flexible ensembles, to select predictors of general schoolwork anxiety in PISA 2015 (Immekus et al., 2022), and recent PISA 2022 studies of mathematics anxiety in single European countries have retained conventional regression frameworks (Aaviste et al., 2025). To our knowledge, no published study has (a) treated the PISA 2022 mathematics anxiety index as the prediction target of a tuned, cross-validated ML pipeline, (b) interpreted the resulting model with exact SHAP decompositions, or (c) done either in the MENA region, whose adolescents are strikingly absent from the interpretable ML literature in educational psychology.

This study addresses this compound gap. Guided by control-value theory (CVT; Pekrun, 2006), we pooled 47,652 fifteen-year-olds from the six Arab PISA 2022 systems and compared six algorithms in predicting mathematics anxiety from 50 predictors spanning eight conceptual domains. We then subjected the best model to a multi-layered interpretability analysis: global SHAP importance, directional and dependence analyses, gender-stratified decompositions, country-by-country replication, and five pre-specified robustness checks. Four research questions structure the investigation, deliberately parallel in form to recent ML studies of achievement so that the affective and cognitive literatures can speak to one another:

RQ1: How accurately can mathematics anxiety be predicted from questionnaire and achievement data, and how do gradient-boosting, bagging, and linear approaches compare?

RQ2: Which factors contribute most to predicted mathematics anxiety, in which direction, and how do these contributions align with CVT?

RQ3: To what extent are predictor contributions uniform across genders and across the six Arab education systems?

RQ4: What implications follow for educational practice and policy in MENA, and for the theory of mathematics anxiety more broadly?

## 2 Literature review

### 2.1 Theoretical framework: control-value theory

This study is grounded primarily in CVT (Pekrun, 2006), the most comprehensive account of the origins of achievement emotions, complemented by social-cognitive theory (Bandura, 1997) and by cognitive models of the anxiety-performance link (Ashcraft and Krause, 2007; Eysenck et al., 2007). CVT holds that achievement emotions such as anxiety arise from two families of appraisals: *control appraisals*, the student’s sense of being able to master the activity and influence its outcomes, and *value appraisals*, the subjective importance of success or failure. Anxiety is theorized to be most intense when a student attaches high value to an outcome while doubting their capacity to attain it. Crucially, CVT specifies that these appraisals are themselves shaped by the learning environment: instructional quality, autonomy and competence support, feedback practices, social expectations, and relational safety all reach emotion through the appraisal system. Longitudinal work confirms the reciprocal architecture the theory implies, with emotions and achievement mutually reinforcing one another over school years (Pekrun et al., 2017).

CVT therefore supplies a principled map from the PISA 2022 questionnaire to theoretically meaningful predictor domains. Mathematics self-efficacy indices operationalize control appraisals directly (Bandura, 1997); prior achievement indexes the objective competence on which control appraisals are partly built; effort and persistence capture motivated engagement; growth mindset represents beliefs about the malleability of ability that feed control appraisals; disciplinary climate, teacher support, cognitive activation, and task exposure describe the instructional environment; belonging, bullying victimization, safety, and family support describe the social environment; and gender, socioeconomic status, and immigrant background locate students in the structural conditions under which appraisals develop. A ML framework complements CVT precisely because the theory is explicit that these antecedents interact and operate conditionally: flexible models can detect such conditionality without requiring it to be specified in advance. Two clarifications about the role of theory are warranted at the outset. First, CVT serves here as an organizing and interpretive framework: it motivates the selection and grouping of predictors and provides the lens through which the predictive results are read. The study does not estimate the structural or causal relationships the theory specifies, and no analysis reported below should be read as a confirmatory test of CVT’s process model. Second, the mapping from theory to measurement is approximate: the PISA indices operationalize control appraisals well (self-efficacy scales) but contain no direct measure of value appraisals, so only the control side of the theory is represented in the predictor space (see Section 5.10).

A second theoretical consideration concerns the direction of the anxiety–achievement association. The Deficit Theory holds that poor performance breeds anxiety, whereas the Debilitating Anxiety Model holds that anxiety consumes the working-memory resources performance requires (Carey et al., 2016; Ma and Xu, 2004). Longitudinal and behavioral-genetic evidence increasingly favors reciprocal causation (Gunderson et al., 2018; Wang et al., 2014). In a cross-sectional design, this reciprocity cannot be disentangled; we therefore include achievement as a predictor on explicitly agnostic terms, quantify its unique contribution, and verify in robustness analyses that the psychosocial findings do not depend on its inclusion.

### 2.2 Correlates of mathematics anxiety

#### 2.2.1 Competence beliefs

Across cultures, mathematics self-efficacy is among the strongest negative correlates of mathematics anxiety, typically exceeding the anxiety–achievement association in magnitude (Lee, 2009; Luttenberger et al., 2018). Meta-analytic estimates place the anxiety–performance correlation around *r* = −0.28 to −0.34 for adolescents (Barroso et al., 2021; Namkung et al., 2019), whereas self-efficacy and related competence beliefs frequently correlate with anxiety at −0.4 to −0.6 (Hembree, 1990; Ma, 1999). Reciprocal models suggest self-concept and anxiety shape one another over time (Ahmed et al., 2012). PISA’s distinction in 2022 between self-efficacy for formal and applied mathematics and self-efficacy for mathematical reasoning and 21st-century tasks permits a more differentiated test of whether the competence–anxiety link generalizes across task types.

#### 2.2.2 Achievement

The negative anxiety–achievement association is among the most replicated findings in educational psychology, holding across more than 60 systems in PISA 2012 (OECD, 2013) and confirmed at the individual and country levels in multilevel analyses of over half a million students (Lau et al., 2022). Its magnitude varies systematically: it is stronger in higher-performing systems and weaker where average performance is low, a moderation relevant to MENA, where achievement distributions sit well below the OECD mean.

#### 2.2.3 Motivation, engagement, and mindset

CVT predicts that motivated engagement and beliefs about the malleability of ability shape emotions through control appraisals. Students who believe intelligence is fixed interpret difficulty as diagnostic of inability, amplifying threat; growth mindset, by contrast, buffers negative emotion under challenge (Yeager and Dweck, 2020). Cross-national PISA analyses associate growth mindset with lower fear of failure and better performance, though effects vary across cultures (Claro et al., 2016; OECD, 2021). Persistence and effort occupy an ambiguous position: engagement is protective in motivational accounts, yet time-intensive studying can also index compensatory struggle by students who find mathematics threatening, predicting higher rather than lower anxiety once competence is held constant. Large-scale evidence on this conditional pattern is limited, and it is exactly the kind of relationship that flexible models are well positioned to reveal.

#### 2.2.4 Instructional environment

Orderly, well-managed mathematics classrooms are associated with lower student anxiety, presumably because predictability supports perceived control (Frenzel et al., 2007; OECD, 2013). Teacher support shows more mixed associations, sometimes reflecting compensatory attention to struggling students in cross-sectional data. Cognitive activation and exposure to demanding reasoning tasks embody a theoretical tension: challenge fosters competence over time, yet, at a given moment, confronting tasks beyond one’s perceived capability may elevate anxiety, especially where instruction is examination-driven. Evidence on these exposure effects for affective outcomes in non-Western systems is scarce.

#### 2.2.5 Social relationships

School belonging correlates with a broad range of positive emotional outcomes (Allen et al., 2018), and bullying victimization with elevated anxiety and depression in and beyond school (Moore et al., 2017). Peer victimization is salient in the MENA systems, which report among the highest bullying exposure rates in PISA (OECD, 2023). Family support functions as a relational resource, though parents can also transmit mathematics anxiety directly, particularly when highly involved (Maloney et al., 2015).

#### 2.2.6 Gender

Girls report higher mathematics anxiety than boys in most systems, a gap only partly explained by achievement differences (Devine et al., 2012; Else-Quest et al., 2010). Habitual self-report measures may overstate the gap relative to real-time experience (Goetz et al., 2013), and its size varies markedly across countries, being paradoxically larger in nominally more gender-equal societies (Stoet et al., 2016). MENA offers a critical test: girls’ achievement equals or exceeds that of boys’ in the region, so any female excess in anxiety cannot be attributed to competence deficits. Beyond mean differences, whether the predictors of anxiety operate equivalently for girls and boys, moderation rather than main effects, has rarely been examined at scale.

#### 2.2.7 Socioeconomic and structural factors

Socioeconomic status correlates with anxiety only weakly at the student level once psychological variables are considered, though income inequality amplifies test anxiety gaps across countries (Claes et al., 2024). Grade repetition, truancy, and immigrant background have all been linked to affective outcomes, but their unique contributions relative to proximal psychological factors remain unclear, particularly outside Western samples.

### 2.3 Mathematics anxiety in MENA

Regional evidence, though sparse, signals both elevated levels and distinctive patterning of mathematics anxiety in Arab systems. Early psychometric work documented substantial mathematics anxiety among Emirati eighth-graders (Alkhateeb, 2001), and parental attitudes toward mathematics predict Emirati adolescents’ motivation and self-beliefs (Areepattamannil et al., 2015). In Qatar, mathematics anxiety shapes secondary students’ arts-versus-science track choices, with higher anxiety among Arts-track students (Megreya and Al-Emadi, 2023). System-level reports show anxiety at or above OECD levels across the Arab PISA participants, coexisting with strong regional commitments to STEM-centred economic diversification (El-Kogali and Krafft, 2019; OECD, 2023). Yet no study has systematically compared the predictors of mathematics anxiety across Arab education systems, and none has used methods capable of ranking dozens of candidate factors on a common scale. The region’s combination of high anxiety, atypical gender configuration, high bullying exposure, and steep between-system heterogeneity in wealth (Morocco’s GDP per capita is roughly one-tenth of Qatar’s) makes it an unusually informative setting for testing the generality of anxiety theory.

### 2.4 Machine learning and explainable AI in large-scale assessment research

Tree-based ensemble methods, random forests (Breiman, 2001), AdaBoost (Freund and Schapire, 1997), and the gradient-boosting frameworks XGBoost (Chen and Guestrin, 2016) and LightGBM (Ke et al., 2017), now routinely outperform linear benchmarks in predicting educational outcomes from questionnaire data, and SHAP has become the *de facto* standard for interpreting them (Lundberg and Lee, 2017; Lundberg et al., 2020). Applications to PISA 2022 include achievement prediction in East Asia, where XGBoost explained about 57% of mathematics score variance with self-efficacy as the dominant predictor (Djekourmane et al., 2026; Wu, 2024), and classification of academically resilient students across 79 systems (Cheung et al., 2024). Methodological guidance for such work stresses honest train–test separation, cross-validated tuning, leakage prevention, and attention to the design features of international assessments, including sampling weights and plausible values (Rutkowski et al., 2010; von Davier et al., 2009; Yarkoni and Westfall, 2017).

Against this backdrop, the outcome side of the paradigm remains lopsided. Affective constructs have served mainly as *predictors* of achievement, not as targets in their own right; the penalized regression study of schoolwork anxiety in PISA 2015 (Immekus et al., 2022) remains the nearest antecedent, but it did not employ flexible ensembles, SHAP, or MENA samples. Flipping the target from achievement to anxiety is not a cosmetic change: it asks what the joint predictor space can tell us about where negative mathematics emotion concentrates, and it confronts ML methods with an outcome whose measurement is noisier and whose theoretical antecedents are more social than cognitive.

### 2.5 This study

The study contributes on four fronts. Substantively, it delivers the first interpretable ML account of mathematics anxiety for approximately 48,000 Arab adolescents, ranking 50 theoretically curated predictors on a common scale. Theoretically, it examines whether the predictive evidence is consistent with CVT’s emphasis on the primacy of control appraisals relative to competing structural accounts, and probes gender moderation at the level of individual predictors. Methodologically, it demonstrates an outcome-flip of the established ML–ILSA paradigm under stricter safeguards than are typical in this literature: constituent-item leakage exclusion, fold-embedded preprocessing, cross-validation-based model selection, plausible-value and weighting sensitivity analyses, and transparent reporting of computational constraints. Practically, it identifies which malleable factors are most tightly coupled to anxiety in a region whose governments are investing heavily in mathematics-dependent economic transitions.

## 3 Methods

### 3.1 Data and sample

We used the public-use student questionnaire database of PISA 2022, the eighth cycle of the OECD’s triennial assessment of fifteen-year-olds, downloaded from the official OECD repository (OECD, 2023). PISA employs a two-stage stratified sampling design, schools, then students within schools, yielding nationally representative samples. All six Arab education systems that participated in PISA 2022 were included: the United Arab Emirates (*n* = 24,600), Palestine (*n* = 7,905), Jordan (*n* = 7,799), Qatar (*n* = 7,676), Saudi Arabia (*n* = 6,928), and Morocco (*n* = 6,867), for a pooled initial sample of 61,775 students.

Because the outcome cannot be meaningfully imputed, the analytic sample was defined as all students with an observed value on the mathematics anxiety index: *N* = 47,652 (77.1% of the pooled sample). Outcome missingness (22.9%) reflects both PISA’s rotated questionnaire design and item nonresponse, and it is demonstrably selective rather than ignorable: students without an ANXMAT value scored on average 0.58 SD lower in mathematics (weighted PISA metric: M = 364 vs. 411 points), were more socioeconomically disadvantaged (ESCS standardized difference = −0.36), and were more often boys (35.4% of students with missing outcomes were girls, vs. 55.0% of students with observed outcomes); a logistic model with these three covariates discriminated missing from observed cases with AUC = 0.71. Missingness rates ranged from 16.1% (Saudi Arabia) to 37.4% (Morocco), with achievement-related selectivity present in every system (standardized achievement differences between −0.33 and −0.65). The analytic sample therefore over-represents higher-achieving students and girls, a restriction that bounds the generalizability of absolute anxiety levels; Section 3.8 describes an inverse-probability-weighting sensitivity analysis that addresses this selectivity directly, and Sections 4.5 and 5.10 discuss its implications for cross-system comparison. The study is a secondary analysis of fully anonymized public data and required no additional ethical approval.

### 3.2 Outcome variable

Mathematics anxiety was measured using the PISA 2022 index ANXMAT, a weighted-likelihood-estimate (WLE) scale derived through item response theory from six items (ST292Q01JA-ST292Q06JA) capturing worry about difficulty in mathematics classes, tension when doing mathematics homework, nervousness with mathematics problems, helplessness, worry about poor marks, and anxiety about failing (e.g., ‘I get very tense when I have to do mathematics homework’; 4-point agreement scale). The index is standardized to a mean of 0 and a standard deviation of 1 across the OECD; higher values indicate greater anxiety. The scale descends from the instrument introduced in PISA 2012 and shows satisfactory reliability across systems (OECD, 2024). The OECD scales all questionnaire indices with international item parameters, testing items for country-level misfit and assigning national parameters where required (OECD, 2024); this supports, but does not establish, full measurement invariance across systems. We did not conduct our own invariance testing, and cross-system comparisons in this study are accordingly restricted to the relative ordering of predictors within each system rather than comparisons of latent construct means (see Sections 4.5 and 5.10). In the weighted analytic sample, all six systems scored above the OECD reference mean (from +0.10 in Saudi Arabia to +0.41 in Morocco).

### 3.3 Predictors

Fifty predictors were assembled from the student file under three constraints. First, *leakage exclusion*: the six constituent items of ANXMAT were excluded from the predictor set, as were any of the derived indices that shared items with it. Second, *coverage*: candidate variables missing for more than 30% of the analytic sample were excluded under a rule fixed before modeling; this removed ten variables, including four social–emotional skill indices not administered in these systems (cooperation, perseverance, assertiveness, stress resistance), life satisfaction, emotional control, empathy, self-directed learning efficacy, expected occupational status, and expected education. Third, *theoretical curation*: remaining variables were organized into eight domains derived from CVT: (1) competence and control beliefs, mathematics self-efficacy for formal/applied tasks (MATHEFF) and for reasoning/21st-century tasks (MATHEF21), perceived ease of mathematics, growth mindset (GROSAGR); (2) engagement, effort and persistence in mathematics (MATHPERS), study time, homework time in mathematics, class periods in mathematics and overall; (3) instructional environment, disciplinary climate (DISCLIM), teacher support (TEACHSUP), two cognitive activation indices, exposure to formal/applied tasks (EXPOFA) and to reasoning/21st-century tasks (EXPO21ST); (4) social environment, sense of belonging (BELONG), bullying victimization (BULLIED), feeling safe (FEELSAFE), school safety risks (SCHRISK), student–teacher relationship quality (RELATST), family support (FAMSUP); (5) achievement, the first plausible value in mathematics (PV1MATH), with PV2-PV10 reserved for sensitivity analyses (von Davier et al., 2009); (6) dispositional and creative factors, curiosity, creative self-efficacy, creative school environment, openness to art and reflection, career information seeking; (7) behavioral conduct, truancy, tardiness, extended absence, grade repetition, and out-of-school activities (exercise, paid work, household work); and (8) demographics, gender, grade, age, immigrant background (first- and second-generation dummies), socioeconomic and cultural status (ESCS), home possessions, information and communication technology (ICT) resources, parental occupational status (HISEI) and education (PAREDINT), plus five system dummies (reference: United Arab Emirates). Table 1 reports descriptive statistics; residual predictor missingness after the coverage rule was at most 14.4%. Supplementary Table S1 lists every predictor with its PISA 2022 variable code, official variable label, conceptual domain, and measurement type.

**Table 1:** Descriptive statistics for all predictors and the outcome (analytic sample, unweighted).

Variable	Domain	Mean	SD	% missing
FEMALE	Demographic	0.55	0.50	0.0
GRADE	Demographic	−0.01	0.65	0.0
AGE	Demographic	15.82	0.29	0.0
IMMIG_SECOND	Demographic	0.12	0.32	4.6
IMMIG_FIRST	Demographic	0.22	0.41	4.6
REPEAT	Behavioral	0.13	0.33	0.6
ESCS	Socioeconomic	−0.23	1.15	0.2
HOMEPOS	Socioeconomic	−0.70	1.21	0.0
ICTRES	Socioeconomic	−0.18	1.55	0.2
HISEI	Socioeconomic	56.60	24.78	8.5
PAREDINT	Socioeconomic	14.27	2.81	0.5
SKIPPING	Behavioral	0.42	0.49	1.5
TARDYSD	Behavioral	0.65	0.76	2.0
MISSSC	Behavioral	0.11	0.32	1.1
STUDYHMW	Engagement	6.91	3.09	0.9
EXERPRAC	Behavioral	4.91	3.58	0.8
WORKPAY	Behavioral	1.64	3.15	0.9
WORKHOME	Behavioral	5.71	3.75	0.9
INFOSEEK	Dispositional	0.27	1.12	7.4
MATHEFF	Competence beliefs	−0.44	1.28	1.7
MATHEF21	Competence beliefs	0.20	1.09	2.2
MATHPERS	Engagement	0.09	1.20	1.4
EXPOFA	Instructional	0.22	1.07	2.4
EXPO21ST	Instructional	0.35	1.13	2.7
TEACHSUP	Instructional	0.28	1.20	1.6
DISCLIM	Instructional	0.04	1.08	1.3
COGACRCO	Instructional	0.07	1.18	2.0
COGACMCO	Instructional	0.32	1.13	2.4
MATHEASE	Competence beliefs	0.09	0.28	2.2
MATHMOT	Engagement	0.05	0.22	2.0
ST059Q01TA	Engagement	6.31	6.58	2.6
ST059Q02JA	Engagement	24.23	15.11	4.6
ST296Q01JA	Engagement	2.09	1.32	1.2
BELONG	Social	−0.16	0.94	1.6
BULLIED	Social	−0.17	1.14	1.1
FEELSAFE	Social	−0.03	1.03	0.9
SCHRISK	Social	0.13	1.10	1.3
RELATST	Social	0.03	1.10	10.3
FAMSUP	Social	0.00	1.15	6.5
GROSAGR	Competence beliefs	0.06	1.13	2.6
CURIOAGR	Dispositional	0.12	1.12	14.4
CREATEFF	Dispositional	0.16	1.11	2.5
CREATSCH	Dispositional	0.30	1.21	3.4
OPENART	Dispositional	0.29	0.97	5.6
PV1MATH	Achievement	410.93	90.87	0.0
CNT_JOR	System	0.12	0.32	0.0
CNT_MAR	System	0.09	0.29	0.0
CNT_PSE	System	0.13	0.34	0.0
CNT_QAT	System	0.11	0.32	0.0
CNT_SAU	System	0.12	0.33	0.0
ANXMAT	Outcome	0.19	1.15	0.0

### 3.4 Preprocessing and analytic pipeline

The pooled analytic sample was split into training (80%; *n* = 38,121) and test (20%; *n* = 9,531) partitions, stratified by system, with a fixed random seed (42). All subsequent preprocessing was estimated on training data only and embedded inside the cross-validation loop to prevent information leakage: missing predictor values were imputed with fold-specific medians, a strategy robust to the skewed distributions typical of questionnaire indices, and features were scaled with fold-specific robust scaling based on the interquartile range. Because the outcome is a bounded IRT index, we did not delete outliers in the main analysis; instead, a sensitivity analysis re-estimated the final model after removing the 1% of training observations flagged as multivariate anomalies by an isolation forest. The primary analysis was estimated without sampling weights, a deliberate choice that follows from the study’s objective. Our goal is predictive modeling and predictor ranking within the analytic sample, not population point-estimation of anxiety levels, for which weighted design-based estimators would be mandatory (Rutkowski et al., 2010). Unweighted estimation also preserves comparability with the ML–ILSA literature to which the model comparison speaks, and avoids giving individual high-weight observations disproportionate influence on tree construction. Because weighting can nonetheless matter when systems of very different sample sizes are pooled (Morocco n = 4,296 vs. the United Arab Emirates *n* = 20,441), two weighted specifications are reported alongside the main results: (a) a senate-weighted refit, in which final student weights (W_FSTUWT) are rescaled to sum to a constant (5,000) per system, so that each system contributes equally, applied to both model fitting and evaluation; and (b) an inverse-probability-of-response weighted refit addressing outcome missingness (Section 3.8). Both alter absolute *R*^2^ only marginally and leave the predictor hierarchy unchanged (Section 4.6), indicating that the substantive conclusions are not artefacts of the unweighted specification. Descriptive statistics reported for populations (Table 2) are always senate-weighted.

**Table 2:** Analytic sample by education system (weighted statistics use senate weights).

System	Initial N	Analytic N	% female	Anxiety M (SD)	Math score M	ESCS M
United Arab Emirates	24,600	20,441	51.9	0.14 (1.20)	442	0.31
Jordan	7,799	5,594	58.7	0.23 (1.05)	369	−0.80
Morocco	6,867	4,296	48.7	0.41 (1.00)	373	−1.66
Palestine	7,905	6,162	62.0	0.22 (0.99)	371	−0.90
Qatar	7,676	5,343	55.7	0.19 (1.22)	428	0.14
Saudi Arabia	6,928	5,816	56.3	0.10 (1.21)	393	−0.28
Pooled	61,775	47,652	—	—	—	—

### 3.5 Models and hyperparameter tuning

Six regression algorithms spanning three model families were compared, mirroring recent ML applications to PISA achievement (Djekourmane et al., 2026): ordinary least squares linear regression and elastic net (Zou and Hastie, 2005) as linear benchmarks; random forest (Breiman, 2001) and AdaBoost with shallow tree base learners (Freund and Schapire, 1997) as bagging/boosting baselines; and the gradient-boosting frameworks XGBoost (Chen and Guestrin, 2016) and LightGBM (Ke et al., 2017). Hyperparameters were tuned by randomized search (Bergstra and Bengio, 2012) under 5-fold cross-validation on the training partition, with mean cross-validated *R*^2^ as the selection criterion. Search spaces comprised regularization strength and mixing for elastic net (24 draws); tree depth, leaf size, feature subsampling, and ensemble size for random forest (6 draws); learning rate, ensemble size, and base-learner depth for AdaBoost (6 draws); and learning rate, tree complexity, subsampling, minimum child weight, and L2 regularization for XGBoost (24 draws) and LightGBM (12 draws). All seeds were fixed for reproducibility. Search breadth was scaled according to computational cost, and configurations exceeding a per-fold compute budget were discarded and reported as such in the released code; this transparency measure affected a minority of random-forest draws and no gradient-boosting draws that ranked competitively. Because the initial budgets differed between the two gradient-boosting frameworks (24 draws for XGBoost vs. 12 for LightGBM), the LightGBM search was subsequently extended by 12 additional draws from the same space (independent seed), equalizing the budgets of the two leading frameworks at 24 draws each; results for both the original and the equalized searches are reported in Section 4.2. The full search spaces and the selected configuration of every algorithm are listed in Supplementary Table S4.

### 3.6 Model evaluation

The tuned configuration of each algorithm was refit on the full training partition and evaluated once on the held-out test partition using four complementary metrics: *R*^2^ (proportion of variance explained), root mean squared error (RMSE) and mean absolute error (MAE) in index standard-deviation units, and Spearman’s rho between predicted and observed anxiety (rank fidelity). Model selection was based exclusively on cross-validated performance; the test set served solely for unbiased final assessment.

### 3.7 Interpretability: SHAP analyses

For the selected model, exact SHAP were computed for every test-set student using the TreeSHAP algorithm as implemented natively in XGBoost (prediction contributions), which is mathematically equivalent to the polynomial-time exact procedure of Lundberg et al. (2020). Four layers of interpretation were conducted: (1) global importance, measured as the mean absolute SHAP value per predictor; (2) direction and shape, assessed using the correlation between predictor values and their SHAP values, alongside dependence plots for the leading predictors; (3) gender stratification, mean absolute SHAP values and local dependence slopes estimated separately for girls and boys, with the slope ratio quantifying moderation; and (4) country decomposition, measured as the share of total absolute SHAP attributable to each predictor within each of the six systems, with cross-system consistency summarized by pairwise Spearman correlations of importance rankings. To aid interpretation, SHAP-based rankings were juxtaposed with senate-weighted bivariate correlations. Throughout, marginal association denotes the bivariate relation between a predictor and anxiety ignoring all other variables, whereas conditional association denotes a predictor’s contribution within the fitted model, holding the remaining 49 predictors at their observed values; divergence between the two indicates overlap, suppression, or non-linearity rather than measurement error. Because SHAP attributions divide credit among correlated features, pairwise correlations among the leading predictors are reported in Supplementary Table S2 and considered explicitly when interpreting ranks (Sections 4.3 and 5.10). Uncertainty for the headline gender-stratified slope ratios was quantified with nonparametric bootstrap resampling of test-set students (2,000 replicates; percentile 95% confidence intervals).

### 3.8 Robustness analyses

Five pre-specified analyses probed the stability of results: (a) exclusion of achievement, refitting the final model without PV1MATH to verify that the psychosocial hierarchy is not an artefact of conditioning on performance; (b) plausible-value sensitivity, replacing PV1MATH with each of PV2–PV10 at prediction time, the recommended treatment when a single plausible value serves as a predictor in a non-inferential, predictive context (von Davier et al., 2009): PV1 is used in the main model, and stability across the remaining nine values bounds the imputation variance; (c) weighted re-estimation, as described in Section 3.4; (d) within-system performance, computing test-set *R*^2^ separately per system; and (e) outlier sensitivity via isolation-forest trimming. In response to reviewer feedback, four further analyses were added: (f) an alternative-imputation sensitivity, refitting the final model with mean rather than median imputation; (g) an inverse-probability-of-response weighted refit, in which the probability of an observed outcome was modeled by logistic regression on achievement, ESCS, gender, and system, and students were weighted by the inverse of this probability (truncated at the 99th percentile) to counteract the selective missingness documented in Section 3.1; (h) a school-clustered cross-validation, replacing random student-level folds with grouped folds that keep all students of a school (1,953 schools) in the same fold, to quantify optimism from school-level dependence in the two-stage PISA design; and (i) a fold-paired model comparison, testing the XGBoost–LightGBM difference across the five shared cross-validation folds.

### 3.9 Software

Analyses were conducted in Python 3.10 with scikit-learn 1.7, XGBoost 3.2, LightGBM 4.6, pandas, and pyreadstat; figures were produced with matplotlib. The complete, seeded analysis pipeline, from database download to every number reported here, is released as supplementary code.

## 4 Results

### 4.1 Descriptive statistics

Table 2 summarizes the analytic sample. Weighted mean mathematics anxiety was positive, above the OECD standardization mean, in every system, ranging from +0.10 in Saudi Arabia to +0.41 in Morocco. Weighted mean achievement ranged from 369 to 442 PISA points, well below the OECD average of 472, whereas socioeconomic composition varied widely (weighted mean ESCS from −1.66 in Morocco to +0.31 in the United Arab Emirates), underscoring the structural heterogeneity of the region. Girls reported higher anxiety than boys in five of six systems; the weighted gender gap was largest in the United Arab Emirates (+0.27), Qatar (+0.18), and Morocco (+0.15), and marginally reversed in Jordan (−0.02). Table 1 reports descriptive statistics for all 50 predictors.

### 4.2 Model comparison (RQ1)

**Figure 1:**
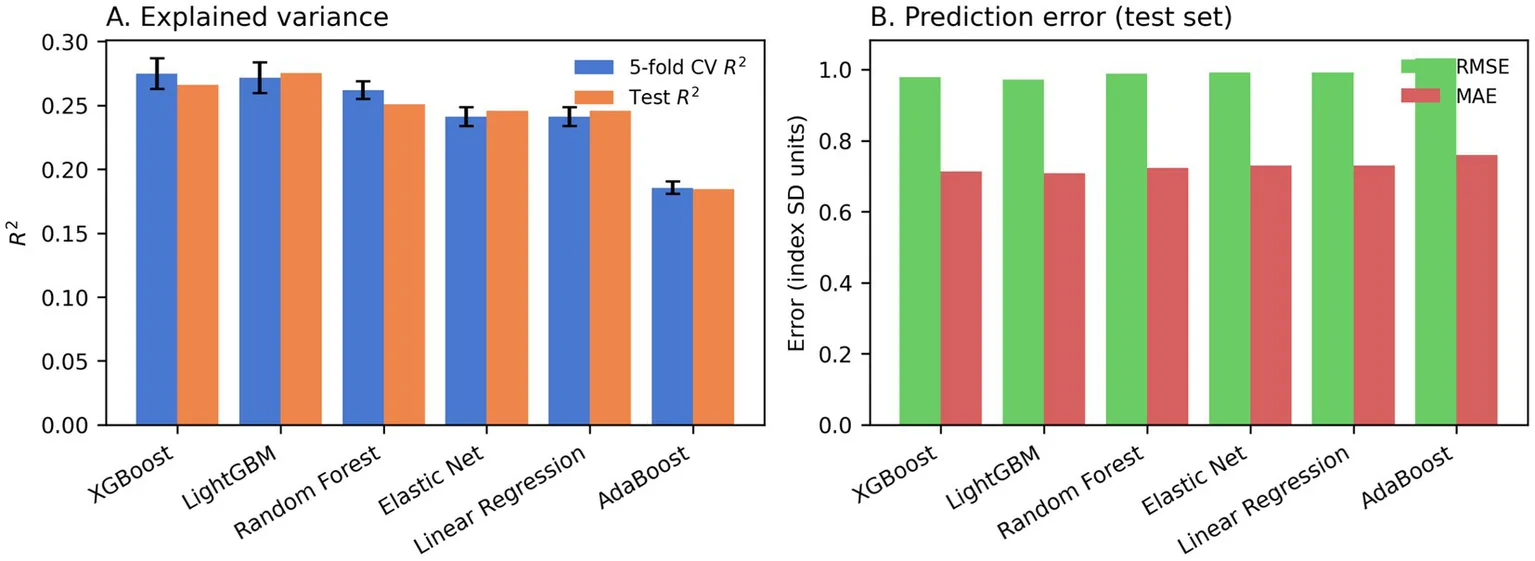
Model comparison: **(A)** cross-validated and test-set *R*^2^; **(B)** test-set prediction error. Gradient-boosting methods outperform bagging and linear benchmarks.

Table 3 and Figure 1 report predictive performance. Model selection followed a rule fixed before any test-set evaluation: the algorithm with the highest mean cross-validated *R*^2^ on the training partition would serve as the final model, and the held-out test partition was accessed exactly once per algorithm, for the final metrics in Table 3; it played no role in tuning or selection. Under this rule XGBoost was selected (CV *R*^2^ = 0.275, SD = 0.012; test *R*^2^ = 0.266, RMSE = 0.978, MAE = 0.712, Spearman’s rho = 0.492). A fold-paired comparison shows that XGBoost’s cross-validation advantage over the originally searched LightGBM configuration, though small (mean difference = 0.003), was consistent across all five folds (*t*(4) = 9.75, *p* = 0.001). On the test set, however, LightGBM performed nominally better (*R*^2^ = 0.275 vs. 0.266), reversing the CV ordering that is within the sampling variability of a single 9,531-student test split. We therefore interpret this result as evidence of practical equivalence between the two frameworks rather than as indicating the superiority of either. After the LightGBM search budget was equalized *post hoc* at 24 draws (Section 3.5), its best configuration reached CV *R*^2^ = 0.276 and test *R*^2^ = 0.279, statistically indistinguishable from XGBoost on the paired folds (*t*(4) = 1.28, *p* = 0.27). Critically, the interpretation does not depend on the choice between the two: the ten most important predictors are identical, in identical order, for XGBoost and for both LightGBM configurations, and the Spearman correlation between the XGBoost and LightGBM importance rankings across all 50 predictors is 0.95–0.98 (Supplementary Table S3). XGBoost is retained as the focal model per the pre-specified rule; all headline conclusions hold verbatim under LightGBM. Random forest followed (test *R*^2^ = 0.251), while the two linear benchmarks explained roughly 24.6% of variance, about 2% points, or roughly one-tenth of explained variance in relative terms, less than XGBoost, indicating meaningful non-additive structure in the predictor–anxiety mapping. The depth-constrained AdaBoost configuration trailed all other learners. The absolute performance ceiling (*R*^2^ about 0.27) is consistent with expectations for affective outcomes measured using short self-report scales, which carry substantially more measurement noise than cognitive scores, and matches the range reported for anxiety-type outcomes in comparable large-scale data (Immekus et al., 2022).

**Table 3:** Model performance: 5-fold cross-validation (training partition) and held-out test partition.

Model	CV R^2^ (M)	CV R^2^ (SD)	Test R^2^	RMSE	MAE	Spearman’s rho
XGBoost	0.2748	0.0119	0.2662	0.9783	0.7123	0.4918
LightGBM	0.2717	0.0120	0.2754	0.9722	0.7076	0.4963
Random forest	0.2618	0.0068	0.2510	0.9884	0.7223	0.4647
Elastic net	0.2412	0.0073	0.2455	0.9920	0.7301	0.4705
Linear regression	0.2411	0.0073	0.2458	0.9918	0.7300	0.4709
AdaBoost	0.1854	0.0049	0.1846	1.0313	0.7594	0.4217

### 4.3 Global predictor importance and direction (RQ2)

**Figure 2:**
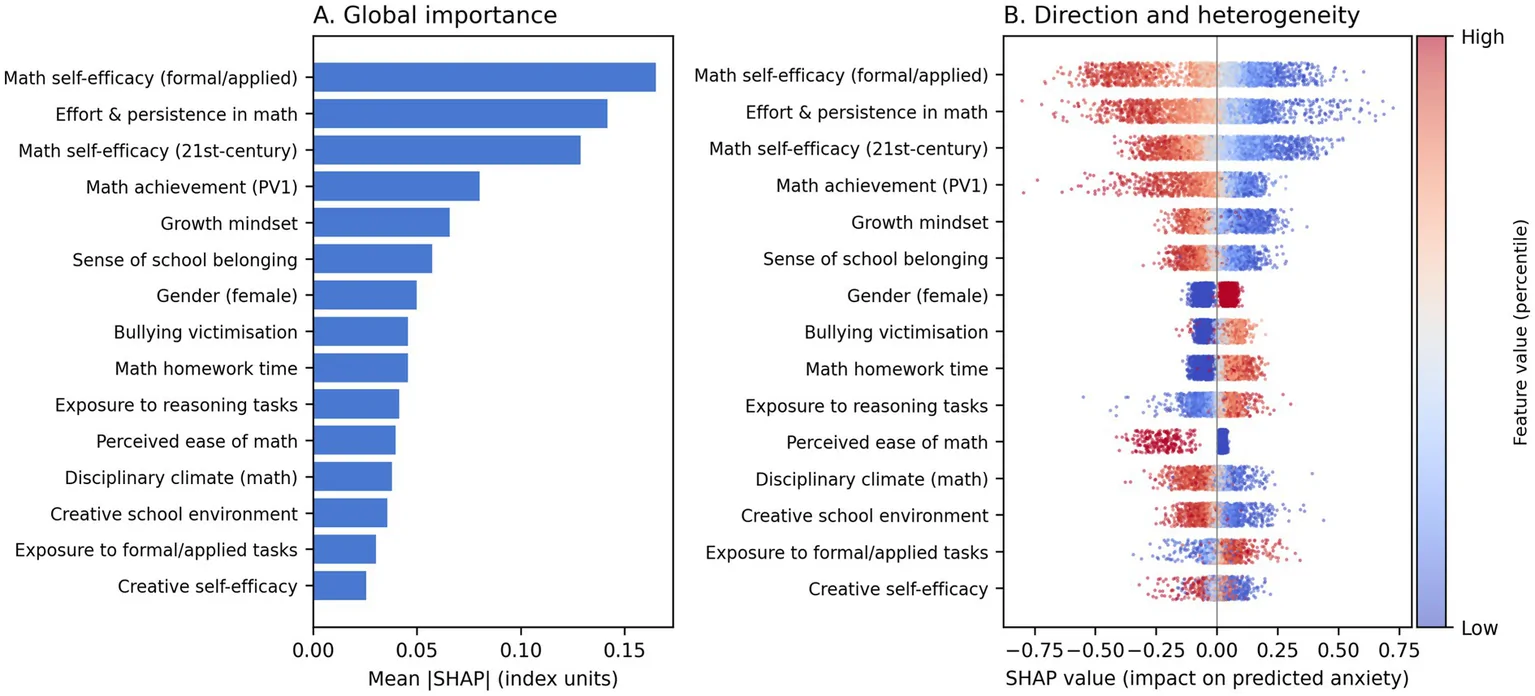
SHAP summary for the final XGBoost model on the held-out test set (*n* = 9,531). **(A)** Mean absolute SHAP value; **(B)** per-student SHAP values: colour encodes the feature value percentile (red high, blue low). Negative SHAP values indicate contributions toward lower predicted anxiety.

Figure 2 presents the SHAP summary; Table 4 quantifies the top 15 predictors. Mathematics self-efficacy for formal and applied tasks dominated (mean |SHAP| = 0.165), followed by effort and persistence in mathematics (0.142) and self-efficacy for mathematical reasoning (0.129); together the three competence-and-engagement indices contributed more predictive weight than the next twelve predictors combined. Mathematics achievement ranked fourth (0.080), followed by growth mindset (0.066), school belonging, gender, bullying victimization, and mathematics homework time. Structural variables were conspicuously weak: socioeconomic status ranked 18th, and no country dummy, parental education, or resource index entered the top 15, indicating that within these systems anxiety concentrates along psychological rather than demographic lines.

**Table 4:** Top 15 predictors of mathematics anxiety: global SHAP importance, direction, and weighted bivariate correlation.

Rank	Predictor	Mean absolute SHAP	Direction r (x, SHAP)	Weighted r with anxiety
1	Mathematics self-efficacy (formal/applied)	0.165	−0.93	−0.367
2	Effort and persistence in mathematics	0.142	−0.95	−0.338
3	Mathematics self-efficacy (reasoning/21st-century)	0.129	−0.92	−0.359
4	Mathematics achievement (PV1)	0.080	−0.90	−0.204
5	Growth mindset	0.066	−0.74	−0.011
6	Sense of school belonging	0.057	−0.80	−0.175
7	Gender (female)	0.050	+0.94	+0.045
8	Bullying victimization	0.046	+0.68	+0.108
9	Mathematics homework time	0.046	+0.75	+0.001
10	Exposure to reasoning/21st-century tasks	0.042	+0.73	+0.004
11	Perceived ease of mathematics	0.040	−0.96	−0.096
12	Disciplinary climate in mathematics	0.038	−0.77	−0.204
13	Creative school environment	0.036	−0.82	−0.274
14	Exposure to formal/applied tasks	0.030	+0.68	+0.020
15	Creative self-efficacy	0.026	−0.44	−0.213

Directional structure (the correlation between a predictor’s values and its SHAP values; Table 4) was theoretically coherent throughout: higher self-efficacy, persistence, achievement, growth mindset, belonging, perceived ease, disciplinary climate, and creative school environment predicted lower anxiety, whereas being female, bullying victimization, longer mathematics homework time, and greater exposure to reasoning-oriented tasks predicted higher anxiety.

Because SHAP attributions divide predictive credit among correlated features, the correlation structure of the leading predictors matters for interpreting ranks. Pairwise correlations among the top 15 predictors were moderate at most (Supplementary Table S2): the two self-efficacy indices correlated at *r* = 0.67, self-efficacy and persistence at *r* = 0.43–0.44, self-efficacy and achievement at *r* = 0.37, and belonging with disciplinary climate at *r* = 0.21; no other pair exceeded |*r*| = 0.45. The most consequential implication concerns the two self-efficacy indices: their shared variance means the model may split efficacy-related credit between them, so their individual ranks are less informative than their joint dominance, and we accordingly interpret them primarily as a single competence-belief block whose combined contribution (mean |SHAP| = 0.29) exceeds that of any other domain. Ranks among weakly correlated predictors (e.g., bullying, homework time, gender) are not materially affected by this mechanism. Comparing conditional (SHAP) and marginal (weighted bivariate) associations exposed two divergences that marginal analyses would miss. Growth mindset was essentially uncorrelated with anxiety bivariately (*r* = −0.011) yet ranked fifth in conditional importance with a consistently negative (anxiety-reducing) direction. A direct check confirms a modest suppression component: the partial correlation between growth mindset and anxiety, controlling for the two self-efficacy indices, persistence, achievement, reasoning-task exposure, and homework time, is *r* = −0.06 (*n* = 44,565), larger in magnitude than the unweighted bivariate *r* = −0.04 in the same complete-case sample. The remainder of growth mindset’s conditional prominence reflects non-linear and subgroup-specific structure that a single partial correlation cannot capture (Section 4.4). We therefore describe growth mindset’s role as a conditional predictive association rather than a demonstrated buffering effect. Mathematics homework time showed the mirror-image pattern (bivariate *r* = 0.001, conditional contribution positive and sizeable), consistent with homework load acting, descriptively, as a marker of anxious, compensatory effort at a given level of competence rather than as a benign engagement indicator.

### 4.4 Dependence structure and gender (RQ3a)

**Figure 3:**
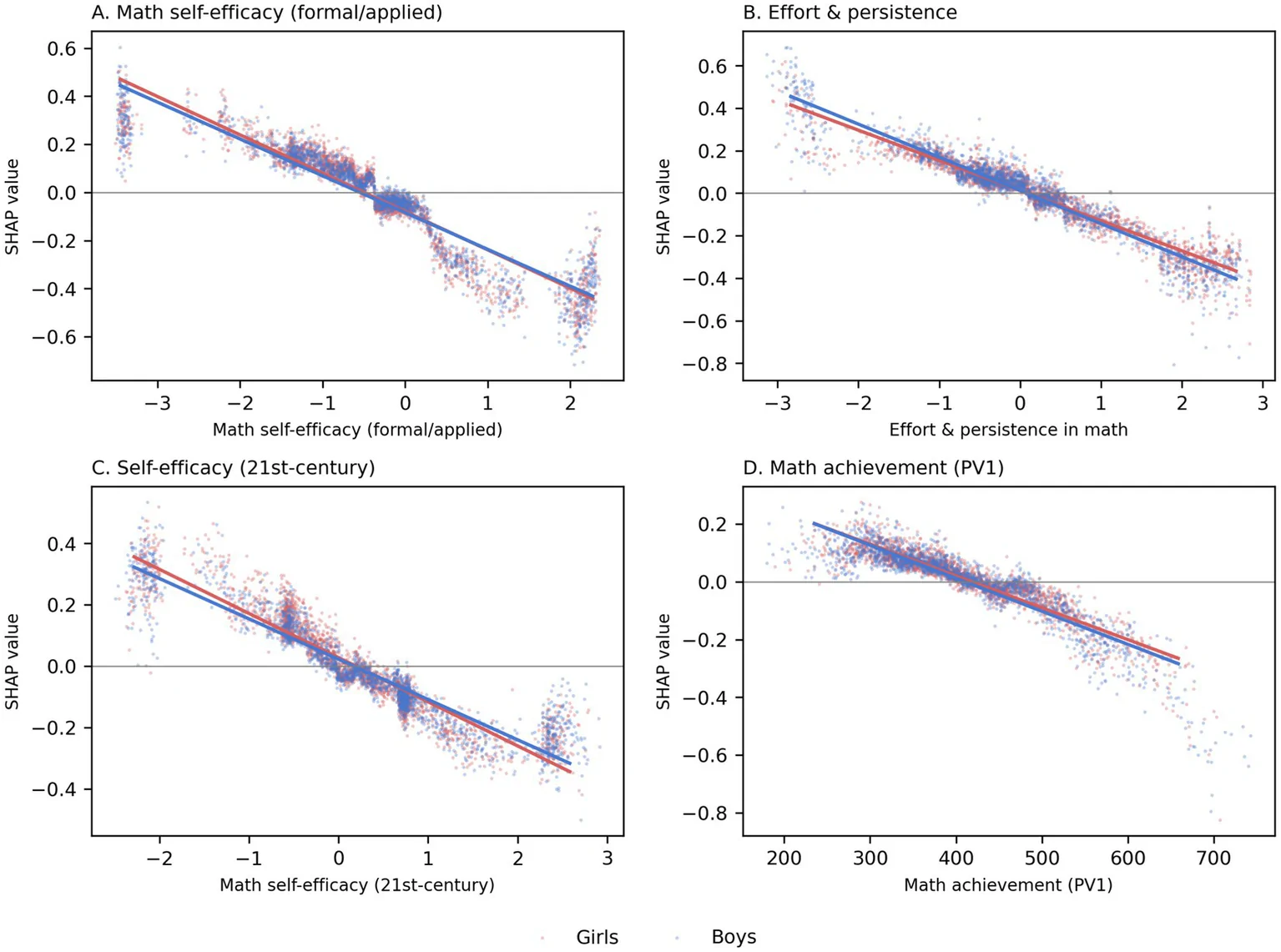
SHAP dependence plots for the four leading predictors, coloured by gender (red = girls, blue = boys), with gender-specific linear fits. (A) Mathematics self-efficacy (formal/applied); (B) effort and persistence in mathematics; (C) mathematics self-efficacy (21st-century); (D) mathematics achievement (PV1). The near-superposition of distributions indicates gender-invariant operation of the competence-engagement core.

Figure 3 shows dependence plots for the four leading predictors, with students coloured by gender. All four displayed steep, monotonic, near-linear gradients with a threshold character around the scale midpoint: students below average self-efficacy or persistence accrued increasingly positive anxiety contributions, while above-average students accrued protective ones. Girls’ and boys’ point clouds were almost perfectly superimposed for the competence-engagement core, with local slope ratios (girls/boys) of 1.04 for formal/applied self-efficacy, 0.91 for persistence, 1.09 for reasoning self-efficacy, and 0.96 for achievement (Table 5): the protective machinery of competence operates essentially identically for both genders.

**Table 5:** Gender-stratified SHAP statistics for selected predictors (test set).

Predictor	Mean abs. SHAP girls	Mean abs. SHAP boys	Slope girls	Slope boys	Slope ratio (G/B)
Mathematics self-efficacy (formal/applied)	0.162	0.169	−0.159	−0.153	1.04
Effort and persistence in mathematics	0.134	0.152	−0.142	−0.156	0.91
Mathematics self-efficacy (reasoning/21st-century)	0.128	0.130	−0.144	−0.132	1.09
Mathematics achievement (PV1)	0.074	0.088	−0.001	−0.001	0.96
Growth mindset	0.074	0.056	−0.067	−0.045	1.50
Sense of school belonging	0.059	0.056	−0.068	−0.060	1.13
Bullying victimiZation	0.049	0.042	+0.043	+0.022	1.92
Mathematics homework time	0.050	0.040	+0.040	+0.026	1.52
Exposure to reasoning/21st-century tasks	0.036	0.049	+0.032	+0.046	0.70
Disciplinary climate in mathematics	0.037	0.039	−0.042	−0.043	0.99

**Figure 4:**
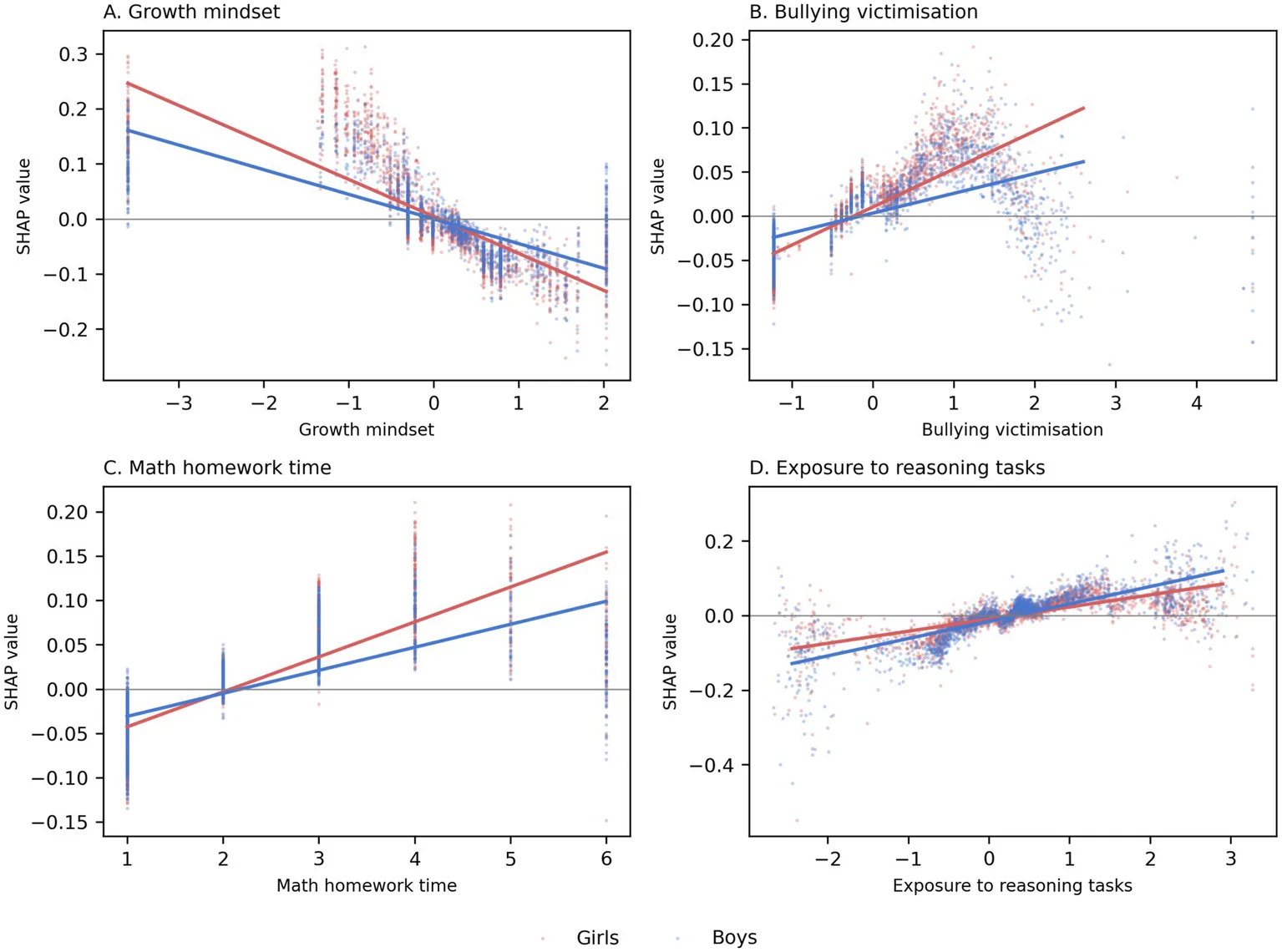
SHAP dependence plots for gender-asymmetric predictors. (A) Growth mindset; (B) bullying victimization; (C) mathematics homework time; (D) exposure to reasoning tasks. The bullying and homework-time gradients are markedly steeper for girls; exposure to reasoning tasks weighs more heavily on boys.

Gender itself, however, carried a systematic main effect: being female shifted predicted anxiety upward by +0.045 index units on average, while being male shifted it downward by −0.056, a net gap of about 0.10 units attributable to gender alone, conditional on all 49 other predictors. Moreover, the social and behavioral periphery was clearly gender-asymmetric (Figure 4; Table 5): the anxiety penalty per unit of bullying victimization was 1.9 times steeper for girls than boys (bootstrap 95% CI [1.75, 2.11]), growth mindset’s negative slope was 1.5 times stronger for girls (CI [1.42, 1.59]), and mathematics homework time was 1.5 times more anxiety-laden for girls (CI [1.42, 1.62]), whereas exposure to reasoning-oriented tasks weighed more heavily on boys (ratio 0.70, CI [0.65, 0.74]). All four intervals exclude 1, indicating that these asymmetries are statistically distinguishable from gender invariance; by contrast, the ratio for formal/applied self-efficacy (1.04, CI [1.01, 1.07]) is significantly but only trivially above 1, supporting the description of the competence core as practically gender-invariant.

### 4.5 Cross-system consistency (RQ3b)

**Figure 5:**
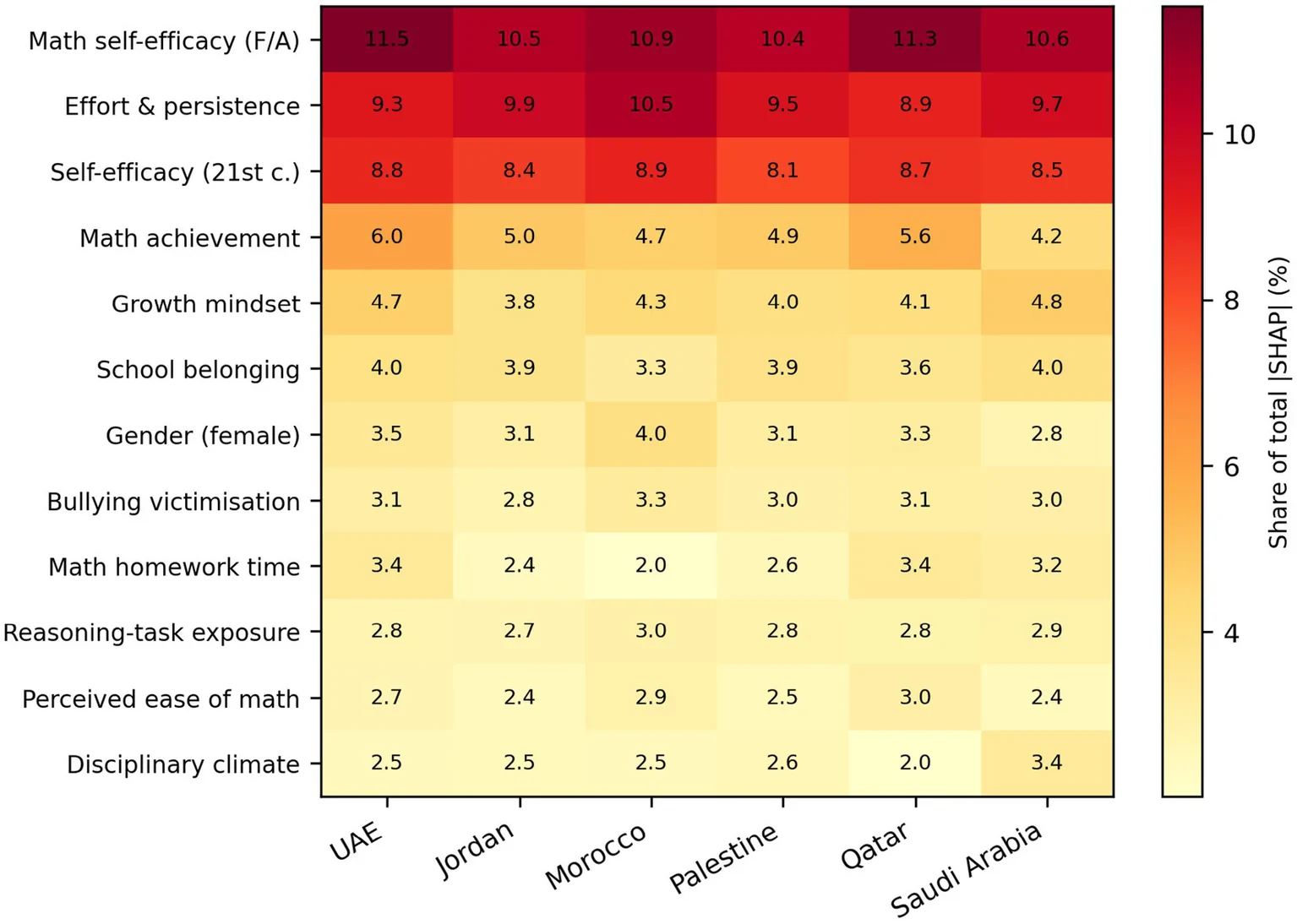
Share of total absolute SHAP contributed by the twelve leading predictors, by education system. For each system, the mean absolute SHAP value of every predictor was computed over that system’s test-set students and divided by the sum of mean absolute SHAP values across all 50 predictors in the same system, so that each column expresses a predictor’s percentage share of that system’s total attributed importance and sums (over all 50 predictors) to 100. The predictor hierarchy is highly consistent across all six Arab systems.

Figure 5 decomposes predictive weight by system. The importance hierarchy replicated across all six systems, mathematics self-efficacy ranked first everywhere (10.4–11.5% of total |SHAP|), and the mean pairwise Spearman correlation between system-specific importance rankings was 0.88 (minimum 0.82), indicating a substantially shared architecture of mathematics anxiety across the region. Absolute predictability nonetheless varied: test-set *R*^2^ was highest in the Gulf systems (Qatar 0.306, United Arab Emirates 0.299, Saudi Arabia 0.264) and lower in Jordan (0.182), Morocco (0.170), and Palestine (0.167), a pattern that tracks the larger anxiety variance in the Gulf samples (weighted SD = 1.20–1.22 in the United Arab Emirates, Qatar, and Saudi Arabia vs. 0.99–1.05 in Palestine, Morocco, and Jordan; Table 2) and plausibly also cross-system differences in response reliability. Two caveats bound this comparison. First, outcome missingness differs across systems (16.1–37.4%; Section 3.1) and is achievement-selective everywhere, so the analytic samples are differentially restricted; the similarity of importance rankings is robust to this concern in direction, but the between-system differences in absolute *R*^2^ partly reflect differential selection as well as substantive differences. Second, formal measurement invariance of the questionnaire indices across the six systems was not established (Section 3.2); the cross-system claims made here therefore concern the ordering of predictors within systems, which is unaffected by system-specific scale shifts, and not comparisons of construct means or absolute effect magnitudes across systems.

### 4.6 Robustness analyses

Table 6 summarizes the five robustness analyses. Excluding achievement reduced test *R*^2^ only from 0.266 to 0.260 and left the top of the hierarchy unchanged (self-efficacy, persistence, reasoning self-efficacy, growth mindset), demonstrating that the psychosocial ranking is not an artefact of conditioning on performance. Substituting plausible values PV2–PV10 for PV1 changed test *R*^2^ by less than 0.002 in either direction (0.2659–0.2677). Senate-weighted re-estimation yielded unweighted test *R*^2^ = 0.260 (weighted *R*^2^ = 0.245) with the same four leading predictors in identical order. Isolation-forest trimming of 1% of training observations (382 students) left performance effectively unchanged (*R*^2^ = 0.264). Four analyses added at review extend this picture. Replacing median with mean imputation changed test *R*^2^ marginally (0.263). The inverse-probability-of-response weighted refit, which up-weights students resembling those lost to outcome missingness (mean weight 1.25), yielded test *R*^2^ = 0.264 (0.261 under IPW-weighted evaluation) with the ten leading predictors identical to the baseline in identical order, indicating that the selective missingness documented in Section 3.1, while real, does not reshape the predictor hierarchy recoverable from the observed sample. School-clustered cross-validation, which prevents students from the same school appearing in both training and validation folds, reduced CV *R*^2^ from 0.275 to 0.259 (SD = 0.013 across folds), quantifying a modest school-level optimism of about 0.016 *R*^2^; predictions for students in entirely unseen schools should therefore be expected to attain *R*^2^ near 0.26 rather than 0.27, with no consequence for the predictor rankings. Finally, the fold-paired XGBoost–LightGBM comparisons are reported in Section 4.2. Together with the per-system replication above, these analyses indicate that the substantive conclusions are stable across every analytic decision we varied.

**Table 6:** Robustness analyses of the final XGBoost model.

Analysis	Specification	Test R^2^	Key result
Baseline	Full model, PV1, unweighted	0.2662	Reference
No achievement	PV1MATH excluded	0.2596	Top-4 psychosocial hierarchy unchanged
Plausible values	PV2–PV10 substituted	0.2659–0.2677	Negligible variation
Sampling weights	Senate-weighted fit and evaluation	0.2603 (unw.) / 0.2452 (w.)	Same leading predictors, same order
Outlier trimming	Isolation forest, 1% removed (*n* = 382)	0.2638	Effectively unchanged
Mean imputation	Mean replaces median (predictors)	0.2631	Effectively unchanged
Nonresponse (IPW)	Inverse-probability-of-response weights	0.2636 (unw.)/0.2612 (IPW)	Top-10 identical to baseline
School-clustered CV	Grouped 5-fold CV by school (1,953 schools)	0.2587 (CV)	About 0.016 *R*^2^ optimism from school overlap; rankings unaffected
Within-system	Separate test *R*^2^ per system	0.167–0.306	Hierarchy replicates; rank rho = 0.88

## 5 Discussion

### 5.1 Principal findings

This study delivered the first interpretable ML account of mathematics anxiety among Arab adolescents, based on 47,652 students from all six MENA systems in PISA 2022. Four findings organize the contribution. First, a tuned XGBoost model predicted anxiety with a test-set *R*^2^ of 0.27, outperforming linear benchmarks by roughly 10% of explained variance and revealing that a meaningful share of the predictor–anxiety mapping is non-additive. Second, the predictive hierarchy was predominantly psychological: two self-efficacy indices and effort–persistence dwarfed every structural, instructional, and demographic variable, with achievement itself ranking only fourth. Third, gender operated in a differentiated way that mean-difference designs cannot capture: a systematic female anxiety surplus conditional on all other predictors, gender-invariant operation of the protective competence core, and gender-asymmetric social stressors, most strikingly a bullying penalty nearly twice as steep for girls. Fourth, this architecture replicated across six systems that differ enormously in wealth, migration profile, and school organization, with importance rankings correlating at 0.88 on average.

It bears emphasizing at the outset that SHAP-based importance quantifies predictive contribution within one model fitted to observational data; it licenses claims about where anxiety concentrates and what would flag risk early, not about what causes anxiety (Yarkoni and Westfall, 2017). With that discipline in place, the pattern of results is nonetheless theoretically pointed.

### 5.2 The primacy of control appraisals

The results are strongly consistent with CVT’s core prediction that emotions in achievement settings arise principally from control appraisals in interaction with subjective value (Pekrun, 2006), while falling short of a test of the theory’s causal process model (Section 2.1). The two self-efficacy indices jointly contributed more predictive weight than the following ten predictors combined, and their SHAP gradients were steep, monotonic, and threshold-like around the scale midpoint: it is below-average confidence, more than above-average confidence, that carries information about anxiety. That the formal/applied and the reasoning/21st-century self-efficacy indices contributed separately and cumulatively suggests that control appraisals are somewhat task-differentiated; students may feel efficacious for procedural mathematics yet threatened by open-ended reasoning, a nuance invisible in cycles that measured self-efficacy unidimensionally.

The dominance of self-efficacy over achievement deserves particular attention. Objective competence (PV1MATH) mattered, but its conditional contribution was half that of perceived competence, and excluding it entirely cost the model only 0.007 *R*^2^ while leaving the psychosocial hierarchy intact. In CVT terms, appraisal mediates reality: what predicts a student’s mathematics anxiety is less what they can do than what they believe they can do. This echoes the East Asian achievement literature in mirror image, where the same self-efficacy index dominated the prediction of performance (Djekourmane et al., 2026; Wu, 2024), a convergence whose practical consequences are developed in Section 5.9. Given the *r* = 0.67 correlation between the two efficacy indices (Supplementary Table S2), we do not over-interpret their individual ranks; the robust claim is the dominance of the competence-belief block as a whole.

### 5.3 Effort, persistence, and the anxious-striving pattern

Effort and persistence in mathematics ranked second, ahead of reasoning self-efficacy and achievement, with higher persistence predicting lower anxiety. At first glance, this simply confirms motivational accounts in which engaged students feel better. Two subtler results complicate the picture. Mathematics homework time predicted higher anxiety conditionally, despite a null bivariate correlation, and exposure to reasoning-heavy tasks did likewise. Holding competence and persistence constant, the student who spends long hours on mathematics homework is disproportionately likely to be an anxious one. This is consistent with a compensatory-effort account: for students whose control appraisals are fragile, additional time on task indexes struggle and threat rather than healthy engagement—the affective counterpart of findings that sheer instructional time yields diminishing returns for achievement in high-intensity systems. The pattern is correlational and cannot establish that assigned homework produces anxiety; the reverse (anxious students taking longer) and third-variable explanations remain fully open. What the data do show is that long mathematics homework time is a marker of elevated anxiety risk at any given competence level, and, per Section 5.5, more strongly so among girls. For policy, this identifies homework load as a visible flag for affective screening and as a candidate target whose causal role warrants experimental evaluation, rather than as a demonstrated lever.

### 5.4 Growth mindset as a conditionally protective belief

Growth mindset presented the study’s clearest demonstration of why flexible models matter for psychological theory. Bivariately, mindset and anxiety were uncorrelated in this sample (*r* = −0.01), a result that, reported alone, would feed scepticism about mindset constructs in non-Western settings. Conditionally, mindset ranked fifth with a consistently protective direction, and its protective slope was 1.5 times steeper for girls. Part of the reconciliation is suppression, directly confirmed in a linear approximation: controlling for competence beliefs, persistence, achievement, and exposure variables raises the (unweighted, complete-case) mindset–anxiety correlation from −0.04 to a partial *r* of −0.06 (Section 4.3). The modest size of this partial coefficient indicates that suppression alone does not account for mindset’s conditional rank; the remainder reflects the non-linear, subgroup-differentiated structure visible in Figure 4, particularly the steeper association among girls. Substantively, viewing ability is malleable appears to defuse the threat value of difficulty, exactly the mechanism proposed by mindset theory (Yeager and Dweck, 2020) and consistent with cross-national PISA evidence linking mindset to lower fear of failure (OECD, 2021; Claro et al., 2016). For a region whose systems are frequently characterized as examination-driven and ability-sorting, the finding that a malleability belief carries an independent negative association with anxiety, particularly for girls, marks mindset-supportive practice as a promising candidate for experimental intervention research rather than a demonstrated lever.

### 5.5 Gender: invariant core, asymmetric periphery

The gender findings refine, and partially challenge, the received view of the gender gap in mathematics anxiety. A female surplus of roughly a tenth of a standard deviation persisted conditional on 49 covariates, in systems where girls match or outperform boys academically, indicating that, within the fitted model, the gap is not attributable to achievement differences (Devine et al., 2012; Else-Quest et al., 2010) and consistent with accounts locating it in gendered socialization of emotional expression and domain identity rather than ability (Goetz et al., 2013; Stoet et al., 2016). More novel is the two-tier moderation structure. The competence-engagement core operated with near-perfect gender symmetry, slope ratios between 0.91 and 1.09, indicating that the primary protective machinery is not gendered. The social periphery was different: bullying victimization was nearly twice as anxiety-laden per unit for girls, and homework burden 1.5 times so, while reasoning-task exposure weighed more on boys. Peer victimization, in other words, appears to translate into mathematics-domain distress far more efficiently for girls, an interaction that, to our knowledge, has not previously been documented at this scale and that connects the bullying literature (Moore et al., 2017) to achievement-emotion research in a specific, testable way. Given that the Arab PISA systems report among the highest bullying exposure rates of all participants (OECD, 2023), the gendered statistical coupling of victimization to mathematics anxiety suggests that anti-bullying policy may also function as mathematics-emotion policy, a hypothesis that intervention studies should now test.

### 5.6 What barely matters: the quiet of structure

As informative as the top of the hierarchy is its bottom. Socioeconomic status, home possessions, ICT resources, parental education and occupation, immigrant background, and the system dummies themselves all contributed marginally, echoing evidence that student-level SES relates only weakly to anxiety once psychological variables are considered (Claes et al., 2024), and extending it to a region with extreme between-system wealth differences. Two readings deserve note. The optimistic reading is that mathematics anxiety in MENA is not structurally locked in: it clusters along malleable psychological lines, within reach of schools. The cautionary reading is that structural factors may operate distally, by shaping the quality of instruction and the formation of competence beliefs; in a student-level cross-section, such distal influences are absorbed into the proximal psychological variables. Both readings agree on the practical point: targeting resources alone is unlikely to move the affective needle.

### 5.7 A shared regional architecture

The near-invariance of the predictor hierarchy across six systems, spanning a tenfold range in per-capita income, majority-national and majority-expatriate student bodies, and post-conflict and high-stability contexts, is among the study’s most policy-relevant results. It implies that the psychological grammar of mathematics anxiety is substantially portable across Arab education systems and, therefore, that intervention models, screening instruments, and teacher-training curricula developed in one system, or pooled regionally through bodies such as ALECSO or the Arab Thought Foundation, have a reasonable prospect of successful transfer. At the same time, absolute predictability was systematically higher in the Gulf (*R*^2^ up to 0.31) than in Jordan, Morocco, and Palestine (0.17–0.18), tracking the compressed anxiety variance in the latter samples; region-wide screening tools should therefore be validated locally even if their ingredient list travels well.

### 5.8 Methodological contributions

Beyond substance, the study demonstrates that the ML-plus-SHAP paradigm now standard for achievement prediction can be flipped to affective targets under stricter safeguards than the literature typically applies: constituent-item leakage exclusion; preprocessing nested entirely within cross-validation folds; model selection by cross-validation with the test set quarantined for a single final assessment; explicit plausible-value and sampling-weight sensitivity analyses in place of the common practice of averaging plausible values and ignoring design weights; per-fold computational budgets reported transparently rather than silently truncating searches; and exact TreeSHAP computed natively rather than approximated. The observed ceiling, roughly 27% of variance, also contributes a calibration point for the field: affective indices measured by six-item self-report scales are predictable to a real but bounded degree, roughly half the variance share achievable for cognitive scores in comparable pipelines, and reviewers of ML-education studies should treat markedly higher affective *R*^2^ values with corresponding suspicion of leakage.

### 5.9 Implications for practice and policy

Three implications follow, framed throughout as screening applications and candidate intervention targets: the evidence is predictive and cross-sectional, and none of the following should be read as a demonstrated causal recommendation until experimental or longitudinal validation exists. First, because low mathematics self-efficacy is the strongest single flag for anxiety, and is measured by short item batteries feasible in routine school assessment, efficacy-focused screening could identify affectively at-risk students far more efficiently than achievement data alone, particularly in the Gulf systems where prediction is strongest. Pedagogically, the efficacy-first pattern favors instruction that manufactures authentic mastery experiences: appropriately calibrated challenge, formative feedback, and structured success, the classical sources of efficacy beliefs (Bandura, 1997). Second, homework and task-exposure policies should be effectively differentiated: identical assignments carry different emotional price tags depending on students’ competence beliefs, and quantity-based prescriptions risk taxing precisely the anxious strivers, disproportionately girls, they aim to help. Third, the gendered bullying–anxiety coupling argues for integrating peer-safety programmes into mathematics-education policy portfolios rather than treating them as separate welfare concerns; in high-victimization systems, a credible anti-bullying programme may be one of the more cost-effective mathematics–anxiety interventions available.

### 5.10 Limitations and future research

Six limitations bound the conclusions. First, the design is cross-sectional; reciprocal dynamics between anxiety, efficacy, effort, and achievement are well documented (Carey et al., 2016; Gunderson et al., 2018; Pekrun et al., 2017), and no ordering claims are made. Longitudinal ILSA linkages or panel studies in the region should test whether the predictive hierarchy identified here anticipates anxiety growth. Second, all predictors and the outcome derive from self-report within a single sitting, so shared method variance inflates psychological associations relative to administrative variables; multi-informant designs would sharpen the comparison. Third, outcome missingness of 22.9% is demonstrably selective, with lower-achieving, lower-SES students and boys under-represented in the analytic sample (Section 3.1), and it varies across systems (16.1–37.4%). The inverse-probability-weighting and mean-imputation sensitivity analyses (Section 4.6) show that the predictor hierarchy is robust to plausible corrections, but absolute anxiety levels and cross-system *R*^2^ comparisons should be read as conditional on the observed sample, and predictor missingness handled by median imputation remains a pragmatic simplification. Fourth, several theoretically relevant constructs, test anxiety, parental mathematics anxiety, teacher emotion, and mathematics value appraisals, are unmeasured in PISA 2022, and their omission likely deflates the explicable variance; the value half of CVT in particular remains untested here. Fifth, although the six systems constitute the entire Arab PISA 2022 cohort, they over-represent the Gulf and exclude most of the Maghreb and Levant; generalization to non-participating Arab systems is conjectural. Sixth, model-agnostic importance is model-dependent in the presence of correlated features: the two self-efficacy indices correlate at *r* = 0.67 and self-efficacy with persistence at *r* = 0.43–0.44 (Supplementary Table S2), so SHAP necessarily divides the competence-belief signal between neighboring indices, and ranks within that block should not be over-read. We mitigated this by reporting bivariate structure alongside SHAP, by interpreting the correlated efficacy indices jointly as a domain block, and by verifying rank stability across model families (identical top-10 under LightGBM), weights, and subsamples, but alternative decompositions could shift specific ranks within tiers. Seventh, formal measurement invariance of the PISA questionnaire scales across the six systems was not tested here; the OECD’s international scaling with country-misfit checks (OECD, 2024) mitigates but does not remove this concern, which is why cross-system conclusions are confined to within-system predictor orderings rather than cross-system comparisons of construct levels. Future work should extend the outcome-flip paradigm to other affective targets in MENA (belonging, fear of failure), exploit TIMSS 2023 for younger cohorts, and, most importantly, pair the observational hierarchy identified here with randomized efficacy-building and anti-bullying interventions to convert predictive leverage into causal knowledge.

## 6 Conclusion

Across six Arab education systems and nearly 48,000 adolescents, mathematics anxiety proved predictable to a meaningful degree, and its predictive anatomy proved strikingly uniform: perceived competence first, engagement second, achievement a distant fourth, structure barely at all, with a female surplus that survives every control and a social periphery, bullying above all, that taxes girls roughly twice as heavily as boys. The results are consistent, at scale and across cultures, with CVT’s emphasis on perceived control; they supply the region’s education systems with a ranked, replicated map of where mathematics anxiety concentrates; and they demonstrate that interpretable ML can serve affective, not only cognitive, educational science. If the map is right, and its arrows are causal, a question only longitudinal and experimental work can settle, then the way to lower the region’s mathematics anxiety runs less through devices, possessions, or hours, and more through what students believe they can do, and how safe they feel among their peers while learning to do it.

## Data Availability

Publicly available datasets were analyzed in this study. These data can be found here: https://www.oecd.org/en/data/datasets/pisa-2022-database.html.
