# Supplementary material for: What predicts adolescents’ mathematics anxiety in the Middle East and North Africa? An interpretable machine learning analysis of six Arab education systems in PISA 2022
Source: Front Psychol. 2026 Sep 9;17:1929157. doi: 10.3389/fpsyg.2026.1929157 (PMC13599571; doi:10.3389/fpsyg.2026.1929157)
Supplement: Supplementary file 1 [file Supplementary_file_1.DOCX]

**Supplementary Material**

*What predicts adolescents' mathematics anxiety in the Middle East and North Africa? An interpretable machine learning analysis of six Arab education systems in PISA 2022*

# Supplementary Table S1. Predictor inventory

All predictors with PISA 2022 variable codes, conceptual domain, measurement type, and official variable label (OECD, 2024). WLE = weighted likelihood estimate.

| **Variable (as analysed)** | **PISA code** | **Domain** | **Measurement** | **Official label** |
| --- | --- | --- | --- | --- |
| FEMALE | ST004D01T | Demographic | Derived dummy (1 = female) | Student (standardized) gender |
| GRADE | GRADE | Demographic | Simple index/derived | Grade compared to modal grade in country |
| AGE | AGE | Demographic | Simple index/derived | Students' age |
| IMMIG_SECOND | IMMIG | Demographic | Derived dummy from OECD index | Immigrant background (OECD definition) |
| IMMIG_FIRST | IMMIG | Demographic | Derived dummy from OECD index | Immigrant background (OECD definition) |
| REPEAT | REPEAT | Behavioural | Simple index/derived | Grade repetition |
| ESCS | ESCS | Socioeconomic | Simple index/derived | Index of economic, social and cultural status |
| HOMEPOS | HOMEPOS | Socioeconomic | WLE index (IRT) | Home possessions |
| ICTRES | ICTRES | Socioeconomic | WLE index (IRT) | ICT Resources |
| HISEI | HISEI | Socioeconomic | Simple index/derived | Highest parental occupational status (ISEI) based on 4-digit human coded ISCO |
| PAREDINT | PAREDINT | Socioeconomic | Simple index/derived | Index highest parental education (international years of schooling scale) |
| SKIPPING | SKIPPING | Behavioural | Simple index/derived | Skipping classes or days of school |
| TARDYSD | TARDYSD | Behavioural | Simple index/derived | Arriving late for school stricter definition |
| MISSSC | MISSSC | Behavioural | Simple index/derived | Missing school for more than 3 months |
| STUDYHMW | STUDYHMW | Engagement | Simple index/derived | Studying for school or homework before or after school |
| EXERPRAC | EXERPRAC | Behavioural | Simple index/derived | Exercise or practice a sport before or after school |
| WORKPAY | WORKPAY | Behavioural | Simple index/derived | Working for pay before or after school |
| WORKHOME | WORKHOME | Behavioural | Simple index/derived | Working in household/take care of family members before or after school |
| INFOSEEK | INFOSEEK | Dispositional | WLE index (IRT) | Information seeking about future careers |
| MATHEFF | MATHEFF | Competence beliefs | WLE index (IRT) | Mathematics self-efficacy: formal and applied mathematics - response options reversed in 2022 |
| MATHEF21 | MATHEF21 | Competence beliefs | WLE index (IRT) | Mathematics self-efficacy: mathematical reasoning and 21st century skills |
| MATHPERS | MATHPERS | Engagement | WLE index (IRT) | Effort and Persistence in Mathematics |
| EXPOFA | EXPOFA | Instructional | WLE index (IRT) | Exposure to Formal and Applied Mathematics Tasks |
| EXPO21ST | EXPO21ST | Instructional | WLE index (IRT) | Exposure to Mathematical Reasoning and 21st century mathematics tasks |
| TEACHSUP | TEACHSUP | Instructional | WLE index (IRT) | Mathematics Teacher Support |
| DISCLIM | DISCLIM | Instructional | WLE index (IRT) | Disciplinary climate in mathematics |
| COGACRCO | COGACRCO | Instructional | WLE index (IRT) | Cognitive activation in mathematics: Foster reasoning Version B |
| COGACMCO | COGACMCO | Instructional | WLE index (IRT) | Cognitive activation in mathematics: Encourage mathematical thinking Version B |
| MATHEASE | MATHEASE | Competence beliefs | Simple index/derived | Perception of Mathematics as easier than other subjects |
| MATHMOT | MATHMOT | Engagement | Simple index/derived | Motivation to do well in mathematics |
| ST059Q01TA | ST059Q01TA | Engagement | Questionnaire item (ordinal) | Number of [class periods] per week in mathematics |
| ST059Q02JA | ST059Q02JA | Engagement | Questionnaire item (ordinal) | Total number of [class periods] per week for all subjects, including mathematics |
| ST296Q01JA | ST296Q01JA | Engagement | Questionnaire item (ordinal) | How much time spent on homework in: Mathematics homework |
| BELONG | BELONG | Social | WLE index (IRT) | Sense of belonging |
| BULLIED | BULLIED | Social | WLE index (IRT) | Being bullied |
| FEELSAFE | FEELSAFE | Social | WLE index (IRT) | Feeling safe |
| SCHRISK | SCHRISK | Social | WLE index (IRT) | School safety risks |
| RELATST | RELATST | Social | WLE index (IRT) | Quality of student-teacher relationships |
| FAMSUP | FAMSUP | Social | WLE index (IRT) | Family support |
| GROSAGR | GROSAGR | Competence beliefs | WLE index (IRT) | Growth Mindset |
| CURIOAGR | CURIOAGR | Dispositional | WLE index (IRT) | Curiosity (agreement) |
| CREATEFF | CREATEFF | Dispositional | WLE index (IRT) | Creative self-efficacy |
| CREATSCH | CREATSCH | Dispositional | WLE index (IRT) | Creative school and class environment |
| OPENART | OPENART | Dispositional | WLE index (IRT) | Openness to Art and Reflection TBD |
| PV1MATH | PV1MATH | Achievement | Plausible value (IRT) | Plausible Value 1 in Mathematics |
| CNT_JOR | CNT | System | System dummy (ref.: United Arab Emirates) | Country code |
| CNT_MAR | CNT | System | System dummy (ref.: United Arab Emirates) | Country code |
| CNT_PSE | CNT | System | System dummy (ref.: United Arab Emirates) | Country code |
| CNT_QAT | CNT | System | System dummy (ref.: United Arab Emirates) | Country code |
| CNT_SAU | CNT | System | System dummy (ref.: United Arab Emirates) | Country code |
| ANXMAT | ANXMAT | Outcome | WLE index (IRT) | Mathematics Anxiety |

# Supplementary Table S2. Pairwise Pearson correlations among the 15 leading predictors

Analytic sample, pairwise complete observations. Lower triangle shown; variable codes as in Supplementary Table S1. Column headings (1)–(14) correspond to the numbered variables in the row labels.

| Variable | (1) | (2) | (3) | (4) | (5) | (6) | (7) | (8) | (9) | (10) | (11) | (12) | (13) | (14) |
| --- | --- | --- | --- | --- | --- | --- | --- | --- | --- | --- | --- | --- | --- | --- |
| (2) MATHPERS | 0.43 |  |  |  |  |  |  |  |  |  |  |  |  |  |
| (3) MATHEF21 | 0.67 | 0.44 |  |  |  |  |  |  |  |  |  |  |  |  |
| (4) PV1MATH | 0.37 | 0.25 | 0.23 |  |  |  |  |  |  |  |  |  |  |  |
| (5) GROSAGR | -0.02 | -0.06 | -0.04 | 0.12 |  |  |  |  |  |  |  |  |  |  |
| (6) BELONG | 0.18 | 0.20 | 0.18 | 0.11 | -0.01 |  |  |  |  |  |  |  |  |  |
| (7) FEMALE | -0.03 | 0.08 | -0.05 | -0.02 | -0.06 | -0.01 |  |  |  |  |  |  |  |  |
| (8) BULLIED | -0.08 | -0.11 | -0.06 | -0.15 | -0.03 | -0.28 | -0.14 |  |  |  |  |  |  |  |
| (9) ST296Q01JA | 0.04 | 0.08 | 0.04 | -0.02 | -0.04 | -0.04 | -0.00 | 0.11 |  |  |  |  |  |  |
| (10) EXPO21ST | 0.10 | 0.14 | 0.18 | 0.07 | 0.07 | 0.10 | 0.02 | -0.10 | -0.03 |  |  |  |  |  |
| (11) MATHEASE | 0.07 | 0.05 | 0.05 | 0.07 | 0.01 | -0.01 | -0.02 | 0.04 | -0.02 | -0.00 |  |  |  |  |
| (12) DISCLIM | 0.23 | 0.27 | 0.21 | 0.20 | -0.00 | 0.21 | 0.14 | -0.28 | -0.01 | 0.04 | -0.01 |  |  |  |
| (13) CREATSCH | 0.36 | 0.44 | 0.41 | 0.12 | -0.09 | 0.27 | 0.05 | -0.15 | 0.04 | 0.16 | -0.01 | 0.29 |  |  |
| (14) EXPOFA | 0.10 | 0.09 | 0.10 | 0.06 | 0.08 | 0.08 | 0.02 | -0.08 | -0.04 | 0.56 | 0.01 | 0.00 | 0.10 |  |
| (15) CREATEFF | 0.36 | 0.38 | 0.39 | 0.11 | -0.09 | 0.20 | 0.06 | -0.06 | 0.06 | 0.09 | -0.05 | 0.19 | 0.48 | 0.05 |

# Supplementary Table S3. SHAP importance under XGBoost vs LightGBM

Mean absolute SHAP values and ranks for the 15 leading predictors, computed on the held-out test set for the selected XGBoost model and the originally searched LightGBM model. Spearman correlation between the two importance rankings across all 50 predictors: rho = 0.98. The equalised-budget LightGBM configuration (CV R² = 0.276; test R² = 0.279) reproduces the identical top-10 in identical order (rank rho vs XGBoost across all 50 predictors = 0.95).

| **Predictor** | **Mean abs. SHAP (XGBoost)** | **Rank (XGBoost)** | **Mean abs. SHAP (LightGBM)** | **Rank (LightGBM)** |
| --- | --- | --- | --- | --- |
| MATHEFF | 0.165 | 1 | 0.157 | 1 |
| MATHPERS | 0.142 | 2 | 0.141 | 2 |
| MATHEF21 | 0.129 | 3 | 0.131 | 3 |
| PV1MATH | 0.080 | 4 | 0.069 | 4 |
| GROSAGR | 0.066 | 5 | 0.064 | 5 |
| BELONG | 0.058 | 6 | 0.057 | 6 |
| FEMALE | 0.050 | 7 | 0.049 | 7 |
| BULLIED | 0.046 | 8 | 0.046 | 8 |
| ST296Q01JA | 0.046 | 9 | 0.042 | 9 |
| EXPO21ST | 0.042 | 10 | 0.040 | 10 |
| MATHEASE | 0.040 | 11 | 0.040 | 11 |
| DISCLIM | 0.038 | 12 | 0.034 | 12 |
| CREATSCH | 0.036 | 13 | 0.033 | 13 |
| EXPOFA | 0.030 | 14 | 0.025 | 14 |
| CREATEFF | 0.026 | 15 | 0.017 | 17 |

# Supplementary Table S4. Hyperparameter search spaces and selected configurations

Randomized search (Bergstra and Bengio, 2012) under five-fold cross-validation; scoring = mean CV R²; all random seeds fixed (data split 42; search stream 2026; LightGBM budget-equalisation extension stream 2027). Runtime caps applied within the executed environment are noted; they are documented in the released code and affected no configuration that ranked competitively.

| **Algorithm** | **Draws** | **Search space** | **Selected configuration** |
| --- | --- | --- | --- |
| Linear regression | 1 | (no hyperparameters) | — |
| Elastic net | 24 | alpha  ^log-uniform [1e-4, 10]; l1_ratio^  uniform [0, 1] | alpha = 0.0011; l1_ratio = 0.45 |
| Random forest | 6 | n_estimators in {200, 300}; max_depth in {None, 10, 16, 24}; min_samples_leaf in {1, 2, 5, 10}; max_features in {0.3, 0.5, sqrt}; runtime caps: n_estimators <= 200, depth <= 16, min_samples_leaf >= 2; 11 of 30 fold-evaluations (combos 3-5) exceeded budget and were excluded | n_estimators = 300 (capped 200); max_depth = 10; min_samples_leaf = 1 (capped 2); max_features = 0.5 |
| AdaBoost | 6 | n_estimators in {100, 200, 300}; learning_rate in {0.03, 0.1, 0.3, 1.0}; base depth in {3, 4, 5}; runtime caps: n_estimators <= 80, depth <= 3 | n_estimators = 300 (capped 80); learning_rate = 0.3; depth = 4 (capped 3) |
| XGBoost | 24 | n_estimators in {300, 500, 800}; learning_rate in {0.03, 0.05, 0.1}; max_depth in {4, 6, 8}; subsample in {0.7, 0.9, 1.0}; colsample_bytree in {0.7, 0.9, 1.0}; min_child_weight in {1, 5, 10}; reg_lambda in {1, 5} | n_estimators = 800; learning_rate = 0.05; max_depth = 6; subsample = 0.9; colsample_bytree = 1.0; min_child_weight = 1; reg_lambda = 5 |
| LightGBM | 12 + 12 | n_estimators in {300, 500, 800}; learning_rate in {0.03, 0.05, 0.1}; num_leaves in {31, 63, 127}; max_depth in {-1, 8, 12}; subsample in {0.7, 0.9, 1.0}; colsample_bytree in {0.7, 0.9, 1.0}; min_child_samples in {10, 20, 40}; reg_lambda in {0, 1, 5} | original: n_estimators = 500; learning_rate = 0.03; num_leaves = 63; max_depth = 12; subsample = 0.9; colsample_bytree = 0.7; min_child_samples = 10; reg_lambda = 1. Equalised-budget best: n_estimators = 500; learning_rate = 0.03; num_leaves = 31; max_depth = -1; subsample = 1.0; colsample_bytree = 0.7; min_child_samples = 20; reg_lambda = 5 |
